# SARS-CoV-2 virus outbreak and the emergency public health measures in Bosnia and Herzegovina: January – July 2020

**DOI:** 10.17305/bjbms.2020.5081

**Published:** 2021-02

**Authors:** Mirsada Hukic, Mirza Ponjavic, Emin Tahirovic, Almir Karabegovic, Elvir Ferhatbegovic, Maja Travar, Fadila Serdarevic

**Affiliations:** 1 Department of Medical Sciences, Academy of Sciences and Arts of Bosnia and Herzegovina, Sarajevo, Bosnia and Herzegovina; 2 Institute for Biomedical Diagnostic and Research NALAZ, Sarajevo, Bosnia and Herzegovina,; 3 GAUSS Centre for Geospatial Research Sarajevo, Sarajevo, Bosnia and Herzegovina; 4 Faculty of Engineering and Natural Sciences, International Burch University, Sarajevo, Bosnia and Herzegovina; 5 Faculty of Engineering and Natural Sciences, International University of Sarajevo, Sarajevo, Bosnia and Herzegovina; 6 South East European Network for Medical Research (SOVE), Sarajevo, Bosnia and Herzegovina; 7 Epidemic Location Intelligence System (ELIS) Project of Academy of Sciences and Arts of Bosnia and Herzegovina; 8 Faculty of Electrical Engineering, University of Sarajevo, Sarajevo, Bosnia and Herzegovina; 9 Faculty of Medicine, University of Banja Luka, Banja Luka, Bosnia and Herzegovina; 10 University Clinical Centre of the Republic of Srpska, Banja Luka, Bosnia and Herzegovina; 11 Erasmus Medical Center, Erasmus University, Rotterdam, Netherlands; 12 Faculty of Medicine, Sarajevo School of Science and Technology, Sarajevo, Bosnia and Herzegovina

**Keywords:** SARS-CoV-2 outbreak, COVID-19 pandemic, emergency, public health

## Abstract

Between March 5^th^ and July 25^th^, 2020, the total number of SARS-CoV-2 confirmed cases in Bosnia and Herzegovina (BH) was 10,090, corresponding to a cumulative incidence rate of 285.7/100,000 population. Demographic and clinical information on all the cases along with exposure and contact information were collected using a standardized case report form. In suspected SARS-CoV-2 cases, respiratory specimens were collected and tested by real-time reverse-transcriptase polymerase chain reaction assay. The dynamic of the outbreak was summarized using epidemiological curves, instantaneous reproduction number R_t_, and interactive choropleth maps for geographical distribution and spread. The rate of hospitalization was 14.0% (790/5646) in the Federation of Bosnia and Herzegovina (FBH) and 6.2% (267/4299) in the Republic of Srpska (RS). The death rate was 2.2% (122/5646) in FBH and 3.6% in the RS (155/4299). After the authorities lifted mandatory quarantine restrictions, the instantaneous reproduction number increased from 1.13 on May 20^th^ to 1.72 on May 31^st^. The outbreak concerns both entities, FBH and RS, and it is more pronounced in those aged 20-44 years. It is important to develop the communication and emergency plan for the SARS-CoV-2 outbreak in BH, including the mechanisms to allow the ongoing notification and updates at the national level.

## INTRODUCTION

On March 5^th^, 2020, the two first probable cases of SARS-CoV-2 virus reported to the Institute of Public Health (IPH) of Republika Srpska (RS) in Bosnia and Herzegovina (BH) were confirmed positive by real-time reverse-transcription-polymerase chain reaction (RT-PCR) at the University Clinical Center Laboratory of the RS in Banja Luka. The cases were a 35-year-old male returning from Italy and his 14-year-old son. Both experienced mild symptoms of cough, fever, and sore throat. On March 21^st^, the first death occurred in BH. The patient was a 76-year-old woman without a travel history subsequently hospitalized for COVID-19 2 days earlier in Bihac, Federation of Bosnia and Herzegovina (FBH). Enhanced surveillance for COVID-19 cases began on April 25 in the RS and on April 19 in the FBH and revealed many additional cases [1]. This report describes the initial and ongoing SARS-CoV-2 virus outbreak in BH for the period from January to July 2020.

## MATERIALS AND METHODS

### Surveillance

In response to increased reports of SARS-CoV-2 virus infections in BH, from March 5^th^ IPH in RS and FBH issued an entity-wide health alert to health-care providers recommending a collection of a respiratory swab for SARS-CoV-2 virus testing using RT-PCR for persons with influenza-like illness (ILI) and travel history to affected areas. From April 10^th^ in RS and from April 15^th^ in FBH, testing for the SARS-CoV-2 virus was extended to all suspected and probable ILI cases using the WHO criteria, regardless of travel history [2]. Demographic and clinical information on all cases along with exposure and contact information was collected using a standardized case report form. The dynamics of the outbreak were summarized using epidemiological curves, instantaneous reproduction number R_t_, and interactive choropleth maps for geographical distribution and spread [3]. We calculated R_t_ using delayed reporting dates of positive confirmed cases. To estimate the dates of infection, we randomly generated the incubation and symptomatic period (1-6 days). The incubation period was governed by the Weibull distribution [4]. To estimate daily R_t_ and its credible interval pertaining to the previous week, we used EpiEstim R package [3] with the mean and the standard deviation of the serial interval [5].

## RESULTS

**FIGURE 1:**
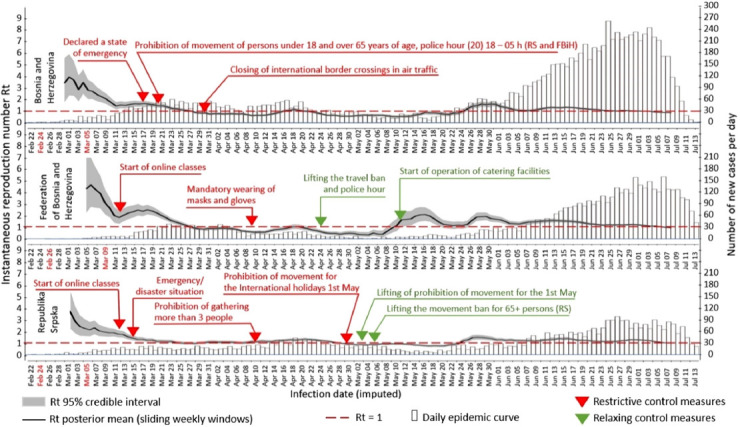
Epidemic curves and weekly Rt estimates based on inferred infection dates.

Between March 5^th^ and July 25^th^, 2020, a total of 4299, 5646, and 145 laboratory-confirmed SARS-CoV-2 cases were reported to IPH of the RS, IPH of the FBH, and Department of Public Health of the District Brčko (DB) starting with 5^th^, 21^st^, and 27^th^ of March respectively, with estimated infection dates between February 24^th^ and July 13^th^, 2020 (Figure 1).

**FIGURE 2:**
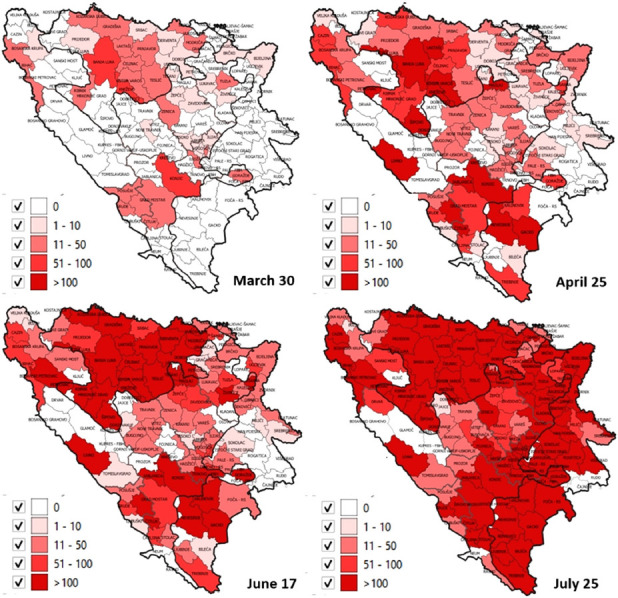
Cumulative number of laboratory-confirmed cases per 100,000 population.

By July 25^th^, 94% of municipalities in FBiH (74/79), 92% of RS municipalities (57/62), and DB reported at least one case (Figure 2). Fifty-one percent of all cases were males.

As shown in Table 1, the majority of cases were 20-44 years old (38.5%). The rate of hospitalization was 14.0% (790/5646) in FBH and 6.2% (267/4299) in RS. The death rate was 2.2% (122/5646) in FBH and 3.6% in the RS (155/4299).

**TABLE 1:**
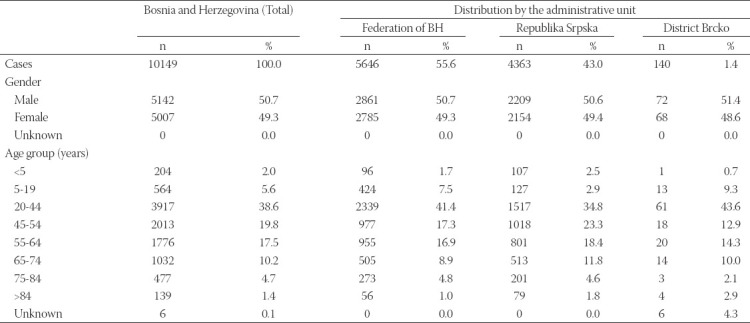
Epidemiologic characteristics of the confirmed cases in Bosnia and Herzegovina

### Prevention and control measures

Beginning March 15^th^, IPH in the RS and March 17^th^ in the FBH issued a number of health alerts to the health care community about infection control, diagnostic testing, and reporting of cases. Early on in the COVID-19 outbreak, they implemented intense measures to facilitate physical distancing, including complete lock-down for children under age 18 years and elderly above age 65 years, curfew, border closings, home-isolation for residents coming from affected areas, obligatory face-masks wearing, and other restrictions in an attempt to slow the spread of the SARS-CoV-2 virus (Table 2) [6,7]. In less than a month, the daily number of new cases in BH leveled off. The instantaneous reproduction number dropped from 1.68 on March 15^th^ to 1.00 on April 15^th^ (Figure 1).

**TABLE 2:**
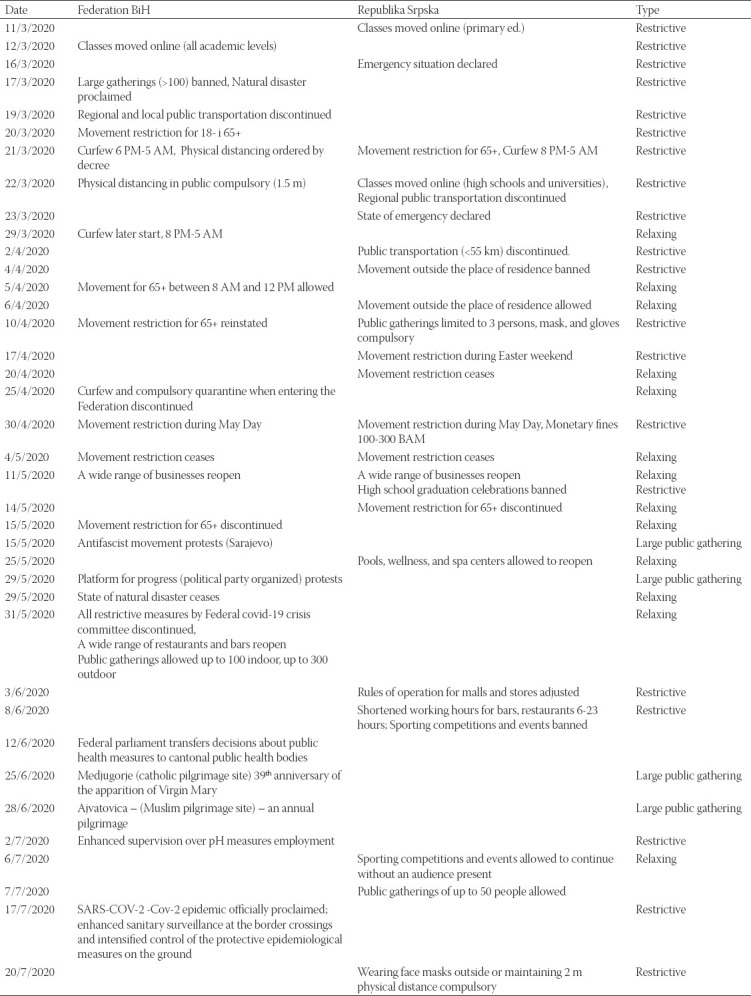
Restrictive, relaxing measures, and large public gatherings in Bosnia and Herzegovina

Until authorities lifted mandatory quarantine restrictions, by April 24^th^ in the FBH and by May 12^th^ in the RS, the number of infected persons was 786 and 985, respectively. Since May 20^th^ (FBH) and May 21^th^ (RS), mandatory self-isolation (14 days) at home upon entry into the country has been abolished. At the same time number of country-wide confirmed cases and instantaneous reproduction number started to increase again from 1.13 on May 20^th^ to 1.72 on May 31^st^ (Figure 1). Despite recommendations to avoid close contact and large congregations, large public gatherings occurred in the country (Table 2). As of July 25^th^, the total number of COVID-19 confirmed cases in BH was 10,090, corresponding to a cumulative incidence rate of 285.7/100,000 population (Figure 1).

Both entities IPH continued recommending mitigation measures, such as wearing face masks, physical distancing, widespread testing for suspected cases, as well as home isolation for residents with acute febrile respiratory illness. They also continued disseminating educational messages regarding respiratory and hand hygiene through the media and their websites (Table 2).

## DISCUSSION

Rapidly establishing surveillance and public health response to the SARS-CoV-2 pandemic was particularly challenging in BH due to the complex organization of the country’s public health and health-care systems.

As a consequence of brutal conflict (1992-1995) that ended up with the Dayton peace agreement, BH has thirteen Ministries of Health (MoH), each with an IPH, for a population of less than 4 million – one for each entity, one for the District of Brčko, and one for each of the ten cantonal ministries in FBiH [8]. IPHs are responsible for communicable disease health monitoring, prevention, and control activities within their administrative jurisdiction. In RS, such regulation and management are under the jurisdiction of the MoH and Social Welfare. The central MoH of FBH coordinates cantonal health administrations at the entity level. The entity-level IPH compiles data and develops monthly/yearly reports for all notifiable diseases. There is limited communication of health data between entities. Communicable diseases data on BH level are compiled by the WHO based on the official entity level IPH monthly reports. This politically driven compartmentalization motivated the Academy of Sciences and Arts of Bosnia and Herzegovina to establish Epidemic Location Intelligence System under the patronage of the BH Presidency to track the SARS-CoV-2 pandemic at the national level.

Lower numbers of confirmed cases of SARS-CoV-2 infections compared to other areas of Europe were reported at the beginning of the outbreak [2]. Possible explanations for the relatively low confirmed case numbers in BH from March 16^th^ until May 20^th^ with average weekly Rt 1.029, and cumulatively 2546 infected individuals can be potentially attributed to early measures, lock-down of the country, border closings, travel measures, home isolation, contact-tracing, and lack of testing. A potential explanation for the subsequent and the current increase in numbers of confirmed cases of SARS-CoV-2 infections from May 21^st^ until July 25^th^ with average weekly Rt for this period 1.19 and cumulatively 7569 infected individuals may be due to discontinuation of obligatory social measures, mass gatherings, and public events, enhanced hospital laboratory capacity and emerged of local transmission. Furthermore, the post-conflict complex governance structure in BH impedes communication and coordination, which in turn poses additional risk for disease spread. For example, when the authorities in the FBH abolished mandatory quarantine at the beginning of April without consultations with the authorities in the RS, it provoked strong reactions from RS authorities who named this “an insane and completely irresponsible move, professionally and epidemiologically unfounded” [9].

Among confirmed cases of SARS-CoV-2 infections in BH, the most affected age group was 20-44 years. These are working-age young people, usually working outside the home and socially most active hence most likely to have attended large gatherings. They can potentially transmit the virus to the elderly and other vulnerable groups later on [10]. The number of reported deaths represents a small percentage, 2.8%, of total confirmed cases, either due to the current high transmission among young people or/and due to lag in transmission and reporting or simply due to expended testing, as more milder cases become identified [11].

### Public health recommendations

Responses for the current wave and preparations for future waves of SARS-CoV-2 influenza infections should be guided by populations that are most likely to be socially active and those at greatest risk for serious complications of SARS-CoV-2 infections. The lockdown of the country at the beginning of the outbreak was reflected in decreased instantaneous reproduction number. However, such a stringent measure is not easily sustainable, as it carries significant psychosocial, economic, and political consequences. Therefore, it is important to develop a communication and emergency plan for SARS-CoV-2 outbreak, including mechanisms to allow ongoing notification and updates at the national level, enable ongoing monitoring of transmission dynamic to facilitate timely adjustments of public health measures, and to communicate risk and event information to all communities including countering misinformation. Emphasis should continue on prompt testing of suspected cases and tracing of their contacts, and on universal prevention measures, including physical distancing, use of face masks, increased hand- and environmental hygiene, and avoidance of mass gatherings and crowded settings [7]. In addition, other strategies proposed recently for developing countries, such as zonal and dynamic blocs, can be considered [12,13]. Finally, coordination and collaboration with neighboring countries and the wider global community will be the key to reducing virus resurgence risks while minimizing travel and trade disruptions.
